# Invariant representations in abstract concept grounding – the physical world in grounded cognition

**DOI:** 10.3758/s13423-024-02522-3

**Published:** 2024-05-28

**Authors:** Jannis Friedrich, Martin H. Fischer, Markus Raab

**Affiliations:** 1 German Sport University Cologne, Germany, Am Sportpark Müngersdorf 6, 50933 Cologne, Germany; 2 Psychology Department, University of Potsdam, Karl-Liebknecht-Strasse 24-25, House 14 D - 14476, Potsdam-Golm, Germany

**Keywords:** Embodiment, Physical invariants, Concepts, Predictive processing

## Abstract

Grounded cognition states that mental representations of concepts consist of experiential aspects. For example, the concept “cup” consists of the sensorimotor experiences from interactions with cups. Typical modalities in which concepts are grounded are: The sensorimotor system (including interoception), emotion, action, language, and social aspects. Here, we argue that this list should be expanded to include physical invariants (unchanging features of physical motion; e.g., gravity, momentum, friction). Research on physical reasoning consistently demonstrates that physical invariants are represented as fundamentally as other grounding substrates, and therefore should qualify. We assess several theories of concept representation (simulation, conceptual metaphor, conceptual spaces, predictive processing) and their positions on physical invariants. We find that the classic grounded cognition theories, simulation and conceptual metaphor theory, have not considered physical invariants, while conceptual spaces and predictive processing have. We conclude that physical invariants should be included into grounded cognition theories, and that the core mechanisms of simulation and conceptual metaphor theory are well suited to do this. Furthermore, conceptual spaces and predictive processing are very promising and should also be integrated with grounded cognition in the future.

## Introduction and motivation

How concepts gain meaning is one of the central questions of the last 50 years in cognitive science (Harnad, 1990; Searle, 1980) and also one of the great targets of embodied and grounded cognition approaches (Fischer & Coello, 2016b; Gallagher, 2011; Shapiro, 2019). These approaches state that cognitive processing takes place not only inside the brain but is inherently body-based. Broadly speaking, grounded cognition research assesses how mental representations of concepts are based in the sensory modalities and action (Barsalou, 2008, 2020). While grounded cognition has focused largely on representations of bodily states (e.g., the perception of a color; Amsel et al., 2014), we propose that representations of physical invariants (called invariant representations) need to be considered to explain the grounding of concepts. Physical invariants fall within the broader category of environmental invariants, and address the physical motion of objects,^1^ for example, gravity, momentum, and friction (cf. below; Hubbard, 1995).

Here we assess the viability of this proposal by reviewing grounded cognition theories’ positions on invariant representations. Our goal is to (1) perform theoretical groundwork for future research, by describing what grounded cognition theories state about our proposal of grounding concepts in invariant representations, and (2) assess the tenability of our proposal by looking at past research and the state of knowledge regarding this topic. We begin with a review of how physical invariants are mentally represented, in order to assess whether they qualify as substrate in which to ground concepts, in the section *Invariant representations*. In the following section, *Concept grounding,* we outline the current consensus on grounding concepts, and then in *Theories of grounded cognition* present the central grounded cognition theories, simulation theory and conceptual metaphor theory, as well as the less commonly cited conceptual spaces and predictive processing theories. In the next section, *Invariant representations in concept grounding*, we discuss their theoretical positions to form the groundwork of future research and empirical positions on invariant representations to assess the current state of knowledge regarding our proposal. Before progressing, we motivate this endeavor with three points.

Firstly, physical invariants should be considered in grounding cognition because they are so fundamental that a research program neglecting their role in cognition is unlikely to gain success. They are the most consistent and ubiquitous features of the environment, being omni-present for every human, both temporally and spatially. Going back to each person’s own birth, our genus’ birth, and our earliest single-celled ancestors, every being’s life is spent adhering to these physical forces. Physical invariants are so pervasive that the human biological system is perfectly attuned to, and even exploits, the physical invariants of our world, from the cellular to the organismal level (Adamopoulos et al., 2021; Rothschild & Lister, 2003). For example, the digestive tract is fine-tuned to earth’s gravity (Yang et al., 2020). Irritable bowel syndrome, an uncomfortable impairment of the digestive system, has been associated with the erroneous perception of higher gravity caused by psychological factors such as anxiety (Spiegel, 2022). This example demonstrates how physiological processes have evolved to be synergistically intertwined with physical forces, and how this is richly interdependent with cognition.

Moving closer to grounded cognition, the field of cognitive science is not unaware of physical invariants; there is a long and diverse history in research on causal perception (Hafri & Firestone, 2021; Michotte, 1963; Zacks & Tversky, 2001) and intuitive physics (Kubricht et al., 2017; McCloskey, 1983; Piaget, 1927). However, these studies are limited to assessing merely how invariants are perceived or represented, not about how they might be exploited in grounding our conceptual system. Yet, some other work has demonstrated that physical invariants are present in more distant domains as well: There is a uni-directional “gravitational advantage” in perception such that participants show more precision for objects accelerating downwards (Bosco et al., 2008; Moscatelli & Lacquaniti, 2011), and gravity is an important component of proprioception (Gallagher et al., 2021). It has also been demonstrated that gravity plays a coordinating role in time perception (Eagleman, 2004; Lacquaniti et al., 2015). The psychological importance of gravity is further suggested by findings on behavior that can change in response to gravity perception, with exploration more likely when upright than supine (Gallagher et al., 2019). Gravity further has an influence on aesthetic judgments (Gallagher & Ferrè, 2018). In this study, participants found vertical lines to be more attractive than tilted lines. Yet, upon manipulating the direction of the gravity vector, this effect disappeared, suggesting the mechanism is not verticality, but rather alignment with the kinetic (gravitational) force on the body. Another example is that physical invariants have been argued to underlie the relatively high-level perception of motion in music (Hubbard, 2017; Larson, 2012). These findings all suggest that representations of physical invariants are not segregated in a separate compartment, but rather that cognition meaningfully exploits this rich and unchanging domain. This further speaks to the point that they have been unduly excluded from possible grounding substrates. Such a core feature of perception and behavior should be a core component of any grounded cognition account alongside other sensorimotor domains. It is unlikely and would be uneconomical if cognition would have access to such representations and not use them to ground concepts, especially knowing that it does exploit them frequently in other domains. Indeed, other authors have also argued for the inclusion of physical invariants as important components of higher-level cognition, including Shepard (1984, 2001) and, recently, Kent (2024). Although their approaches did not involve embedding these into a theory, and are therefore only marginally related, they do further evidence that pursuing this endeavor holds merit. 

Second, it is time to integrate grounded cognition approaches into existing frameworks in cognitive science (e.g., Garcia-Marques & Ferreira, 2011; cf. Kitcher, 1995). The extensive work on causal perception and physical reasoning are instances of domains that have unnecessarily been segregated from grounded cognition. The core tenet of grounded cognition is that, in order to furnish mental representations with semantic meaning, cognition must be meaningfully coupled to experiences of the environment. Invariant representations are one of the most ubiquitous experiences of our environment, so the wealth of research on invariant representations seems a significant oversight. We believe that the integration of work on causal perception and physical reasoning (i.e., invariant representations) with grounded cognition approaches is a promising development for both research programs: For physical reasoning, to gain its fair recognition as an important component of higher cognition (although in artificial intelligence research, this is already the case; Lake et al., 2017; Liu et al., 2024), and for grounded cognition to expand its scope. In short, invariants are universal, so they should smoothly connect to other domains in the broader arena of cognitive science, which benefits grounded cognition by placing it on firmer ground.

Third, grounded cognition must not be left behind by the “ecological turn” in cognitive science and especially in embodied cognition (Fischer, 2024; Rączaszek-Leonardi, 2016). Recent work assessing environmental invariants’ effects on diverse cognitive processes include how they shape learning across the lifespan (Hartley, 2022), how physical reasoning can be the basis of artificial cognition (Aroca-Ouellette et al., 2021; Lake et al., 2017), and even a special issue on concrete constraints on cognition (Borghi et al., 2022b). In artificial intelligence research, there is controversy on the degree to which decidedly un-embodied large-language models reflect the physical world (Taniguchi et al., 2023; Yildirim & Paul, 2024) and use space, time, and physics to reason (Gurnee & Tegmark, 2023; Lake et al., 2017). Artificial video-generation programs like OpenAI’s *Sora* are also purported to have learned underlying physical laws to come up with a human-like intuitive physics engine (Liu et al., 2024; OpenAI, 2024). These examples demonstrate the progression in cognitive science and AI towards increased awareness and assessment of cognition and its important link to the environment. This development has been called for (Van Elk et al., 2010; Wilson & Golonka, 2013) and subsequently welcomed by many theoreticians in the broader embodied cognition community (e.g., Kelty-Stephen et al., 2022; Spivey, 2023). In the context of grounded cognition, Barsalou and colleagues (Barsalou, 2016; Kiefer & Barsalou, 2013) have explicitly called for increased emphasis of the role of the environment in conceptual grounding. We attempt to better prepare grounded cognition for the ecological turn and pull even with cutting-edge work in artificial intelligence with our proposal.

Before introducing the central thesis, on a point of terminology: A lack of continuity in constructs and theory is rampant in grounded cognition (also discussed in Gentsch et al., 2016; Körner et al., 2023), and especially *grounding* and *embodiment* have unclear meanings in the current theoretical debate. We use the term *grounded cognition* to refer to the domain of research concerned with how concepts gain meaning, as done by e.g., Borghi et al. (2023) and Barsalou (2008). This term is intentionally unrestrictive in the assumed grounding sources (Barsalou, 2020), which is especially relevant for our proposal of a grounding modality outside of the body. Further, to describe the research program in cognitive science that emphasizes distinct roles of body and environment in cognitive processing (such research programs have sometimes been called *situated* or *4E* for embodied, embedded, enacted, and extended cognition; see Coello & Fischer, 2016; Newen et al., 2018), we use the term *embodied cognition*, as is convention (Clark, 1998a; Fischer, 2024; Shapiro, 2007). Lastly, when referring to those influences on cognition that originate from constraints of our physical world, we also align with convention, using the term *groundedness* (Fischer, 2012; Körner et al., 2023).

This position comes with some caveats. First, the term *physical invariants* is used to describe the unchanging physical features of objects in movement, and falls within the broader category of *environmental invariants*, which include all features of our environment (e.g., the 24-h circadian rhythm, unidirectional progression of time). In this context, the term *invariant* refers to the unchanging nature of the physical laws that govern these regularities in physical motion. Therefore, while the amount of momentum a given object has may be variable, all objects in movement have momentum, which means that they continue moving in a direction unless stopped by another force (and all objects accelerate downwards at g = 9.82 m/s^2^ when falling). To compare another grounding source: old age looks different in every person, yet no matter the expression of it, it will relate to the concept of old age. Similarly, momentum can be fast or slow but its presence is unchanging. The mental representations of these physical invariants are termed *invariant representations*. Second, we assume the existence of mental representations. While a discussion of physical invariants in non-representationalist frameworks (e.g., Chemero, 2009; Gibson, 1979) is an exciting prospect, it is beyond the scope of the article. Theorists in this tradition may feel at home with conceptual metaphor theory that has been reconciled with the non-representational dynamical systems view of cognition (e.g., Gibbs & Cameron, 2008; also see Raja, 2018; cf. Kelso, 1995; Spivey, 2008). Third, embodied cognition has historically been in opposition to the more widely accepted “sandwich model” approaches of classical Cartesian cognitivism (also called rational, language of thought, modular, sentential, symbolic, computational, etc. approaches). These approaches assume cognition to be a product of transformations on amodal symbols (Fodor, 1983; Pylyshyn, 1984; more recently Quilty-Dunn et al., 2022). We do not discuss these here as these notions would not assign a special place to invariant representations, and therefore a review would bring no added value.

## Invariant representations

In order to qualify as a viable substrate in which to ground concepts, we must establish that physical invariants are perceived and represented directly (cf. Chatterjee, 2010). If invariants are not internalized, merely taking the form of explicit knowledge, the representation of this knowledge would itself require grounding, and the invariants could thereby not ground other knowledge. Therefore, a necessary criterion for our proposal is that invariants’ representations cannot require transformations, and their activation should be immediate and automatic. For example, momentum should not consist of explicit knowledge that an object tends to continue moving when it is in movement, which is then retrieved only during perception. Rather, all moving objects should be represented as continuing their movement. In other words, when we see two objects collide, do we *know* that force is transferred, or do we *see* it (Bertamini, 2002)? As we argue below, this question has been extensively assessed, and we conclude that physical invariants are represented without transformation or abstraction from perception. While this is a widely accepted position, it should be added that there are others, and for an overview we refer to Ludwin-Peery et al. (2021).

Developmental work on physical reasoning lends support to the idea that physical invariants are fundamental components of perception. Infants show signatures of understanding physical forces early in their development, suggesting that physical invariants are primary functions in cognitive development (Baillargeon, 2004; Piaget & Cook, 1952). Physically unstable constellations are recognized as precarious by infants at 12 months (Baillargeon, 1996; Baillargeon et al., 1992). Sensitivity to gravity- and inertia-violating events is present at 7 months (Kim & Spelke, 1992, 1999), and even as early as 2 days (Bardi et al., 2013). These findings highlight that physical invariants are not learned patterns, but rather fundamental and basic aspects of perception.

For the discussion on concept grounding in invariant representations, it is important to refine our definition of physical invariants and elaborate on its two components. In classical mechanics, the description of a moving object includes *kinematics* (the geometric path of the movement) and *kinetics*^2^ (the force causing the movement; Encyclopedia Britannica, 2016). Gravity consists of a *kinematic* direction (down) and a *kinetic* force pulling an object down. Momentum similarly has a kinematic direction (the direction of movement), while also having kinetic force pushing this object in its direction. These two components are related and evidently co-occurring, but form independent parts of an object’s mechanics (Awrejcewicz, 2012; Bertamini, 2002). The direct perception and representation of kinematics is self-evident and trivial, therefore we do not focus on it further in this section (but discuss physical invariant-kinematics as grounding substrate in the section *Past research through the lens of invariant representations*), instead aiming to demonstrate that kinetic force is represented and capable of grounding concepts.

One research domain looking closer at the nature of how kinetic force is represented is causal perception. Empirical work in this field can be traced to Michotte (1963), who suggested that the transfer of force in a launching display was perceived, as opposed to inferred. Recent work found support for the notion that the kinetic relations among objects are represented alongside other visual features like color (Scholl & Tremoulet, 2000). Hafri and Firestone (2021) outlined the necessary conditions for causal relations, including kinetic force, to be perceived immediately and automatically, identifying four perspectives that offer support: First, signatures of causal perception can be directly perceived even after very brief exposures (Firestone & Scholl, 2017; Hafri et al., 2013). Second, there is perceptual adaptation to launching displays, a type of causal event, implying low-level mechanisms being responsible (Kominsky & Scholl, 2020; Rolfs et al., 2013). Third, attributes that are “perceptual” are favored in visual search tasks, which is also true for causal relations (Yang & Wolfe, 2020). Fourth, perceptual processes are more likely to affect other perceptual processes, which is the case in causal relations (Kim et al., 2013; Wright & Dawson, 1994). Therefore, causal relations among objects, including the transferred kinetic force, are “perceived” in the same way as other sensorimotor perception (Firestone & Scholl, 2017; Hafri & Firestone, 2021). If the transfer of kinetic force can be perceived directly, a logical implication is the direct perception of kinetic force itself.

Perhaps the most researched domain into the representation of physical invariants is that of physical reasoning and, within it, intuitive physics. Intuitive physics assesses how humans understand and predict movement in the physical world intuitively (e.g., McCloskey, 1983). Intuitive physics has sometimes focused on errors in human prediction of physical events. For example, participants often incorrectly believe objects on a curvilinear path will continue moving in a curve (McCloskey et al., 1980; McCloskey & Kohl, 1983). Such findings have been found to be the product of experimental demands or generally in line with effective interaction with the world (Mitko & Fischer, 2023; Smith & Vul, 2013; Vicovaro, 2023). Therefore, while predictions of objects’ movements are imperfect under some circumstances, they are nearly optimal for most purposes, and therefore bear little relevance for the current aims. A detailed discussion of this work and of intuitive physics approaches generally would go beyond the scope of this article, so we refer to Kubricht et al. (2017) and J. Fischer and Mahon (2021) for recent overviews and to Sanborn et al. (2013) for a historical summary. These reviews summarize wide empirical support for the notion that physical invariants are represented directly in dedicated representational substrate. In assessing the understanding of physics, internal modeling approaches have also found success. These consistently demonstrate the perception of kinetic force (White, 2012). For example, the prediction of force changes is reflected in anticipatory grip force adjustments (Flanagan & Wing, 1997). In this realm, a prominent recent framework is the simulation theory,^3^ also called physics engine theory (Vicovaro, 2021; Zago et al., 2008). Simulation theory states that, much like video game physics engines, we simulate the physical world and its underlying forces in order to predict upcoming events (Smith & Vul, 2013; Ullman et al., 2017). Therefore, part of this simulation is not just the kinematic direction, but also the kinetic force (Battaglia et al., 2013; Hegarty, 2004; Schwartz, 1999). For example, astronauts who attempt to catch objects do so earlier in 0 *g* than in earth’s 1 *g* field (McIntyre et al., 2001). This claim has also received support from neuroscientific approaches (Kubricht et al., 2017; Pramod et al., 2022), where, for example, functional magnetic resonance imaging (fMRI) studies suggest distinct brain regions for “inputs” into simulations of physical laws (Fischer et al., 2016a; Schwettmann et al., 2019). In short, not only is there convincing evidence that the kinematics and kinetics of physical invariants are represented, they are also considered to be simulated in a rich model of the external world. This synthesis of diverse lines of research points toward a conception of invariants as internalized. It is critical to prevent our proposal from being trivial that the representations of invariants are not merely a subset of other sensorimotor representations, but rather a separate dedicated grounding substrate. The work we survey above triangulates such a view in which physical processes have their own dedicated representations beyond the sensorimotor system. Developmental work highlights the primacy and fundamental nature of physical reasoning, while the fields of causal perception and physical reasoning triangulate a view under which kinetic force is perceived, represented and simulated directly.

## Concept grounding

The goal of this review is to argue for invariant representations as a possible grounding substrate. Before turning to individual grounded cognition theories’ treatment of invariant representations, we begin with an atheoretical overview of what we extracted as consensus opinions in grounded cognition. This includes concept grounding, the distinction of abstract and concrete concepts (including our decision to focus on abstract concepts), and the currently available grounding substrates.

Grounded cognition says that concept representations consist in a non-negligible part of representations of the body^4^ (Barsalou, 2012). This literature argues that cognition evolved to serve action (see point 5 in Wilson, 2002), and subsequently more abstract, higher-level cognition is based on action and action opportunities (Glenberg & Gallese, 2012; Körner et al., 2023; Pezzulo & Cisek, 2016). For example, sensorimotor representations of interactions with an object form the representation of this object concept. Grounded cognition distinguishes between concepts that are concrete (i.e., perceptible via the modalities, e.g., a cup) and others as abstract (i.e., imperceptible, e.g., virtue). Strong grounded cognition accounts say that all concept representations (even abstract concepts) consist of the activation of concrete representations, while some cognitivists would argue that all concepts consist of disembodied representations (for review, see Borghi et al., 2017). Most theorists today agree that concepts are represented to some degree both in concrete and in non-concrete representations (Dove, 2011; Pecher, 2018; Pexman, 2017). Furthermore, concepts are not represented in solely a single modality: Considering the example of the cup, both visual and haptic representations represent this object (Barsalou et al., 2018; Borghi et al., 2023).

Although the meaning of “abstract” and its intricacies have been elaborately discussed elsewhere (Barsalou et al., 2018; Buccino et al., 2019), we define it here for better clarity. Firstly, there are many varieties of abstract concepts (Borghi et al., 2018, 2022a; Desai et al., 2018, and associated in special issue; Villani et al., 2019), but we will treat them as a unitary whole here, as there is no a priori reason to suspect the influence of invariants to differ across these varieties.

Next, it is important to differentiate abstraction and abstractness. Abstraction is the ability to find the common attributes of multiple instances of a category, which then make up a concept (“sense 1” in Barsalou, 2003; “problem 1: generalization” in Dove, 2016). This is, for example, knowing the attributes that make a cup a cup and abstracting these across specific instances to be able to identify new objects belonging to the category cup. Abstractness, on the other hand, comprises concepts like virtue, truth, or freedom, which do not have physically perceivable referents (“sense 6” in Barsalou, 2003; “problem of disembodiment” in Dove, 2016). We mean “abstract concepts” to refer to this latter view, concepts rated high in abstractness (Borghi et al., 2019; Buccino et al., 2019), which is in line with common definitions (Barsalou, 2003; Borghi et al., 2022b; Kiefer & Harpaintner, 2020).

We choose to focus our discussion of the contribution of invariant representations on the grounding of *abstract* concepts. Firstly, because we intend to formulate a strong version of the argument for invariant representations, and abstract concepts are the more ambitious target. Second, because cognition that goes beyond a given situation is one of the most powerful cognitive abilities that humans possess, and therefore a critical challenge for cognitive science (Barsalou, 2016; Borghi et al., 2017; Gilead et al., 2020). Lastly, in order to advance grounded cognition theories, abstract concepts are a critical point, as one of the core challenges of grounded cognition is its (in)ability to account for grounding abstract concepts (Barsalou, 2003; Dove, 2016; Goldinger et al., 2016; Mahon & Caramazza, 2008; Löhr, 2019; which is not to say that amodal symbols approaches do not have this issue, cf. Lupyan & Winter, 2018).

What substrates are concepts grounded in, according to grounded cognition? Firstly, concrete concepts are typically represented in the format of sensorimotor representations in the modalities of sight, hearing, and even taste or smell (Speed & Majid, 2019). For abstract concepts, on the other hand, the main sources of grounding come from interoception and proprioception (Barsalou, 1999, 2008), including the representation of meta-cognitive states (Pezzulo et al., 2013). Common in more recent developments is the import placed on emotion (Kousta et al., 2011; Vigliocco et al., 2014; although see Winter, 2022), language (Dove, 2020; Lupyan, 2012; Zwaan, 2014), social interaction (Barsalou et al., 2003; Reinboth & Farkaš, 2022), or a combination of both, such as the *Words as Social Tools* approach (Borghi et al., 2019; Borghi & Cimatti, 2009). Different abstract concepts differ in the degree to which they are grounded in these modalities (Lynott et al., 2020; Villani et al., 2019). Nonetheless, even abstract concepts can be grounded in sensorimotor representations (Fischer, 2012; Harpaintner et al., 2020).

**Fig. 1:**
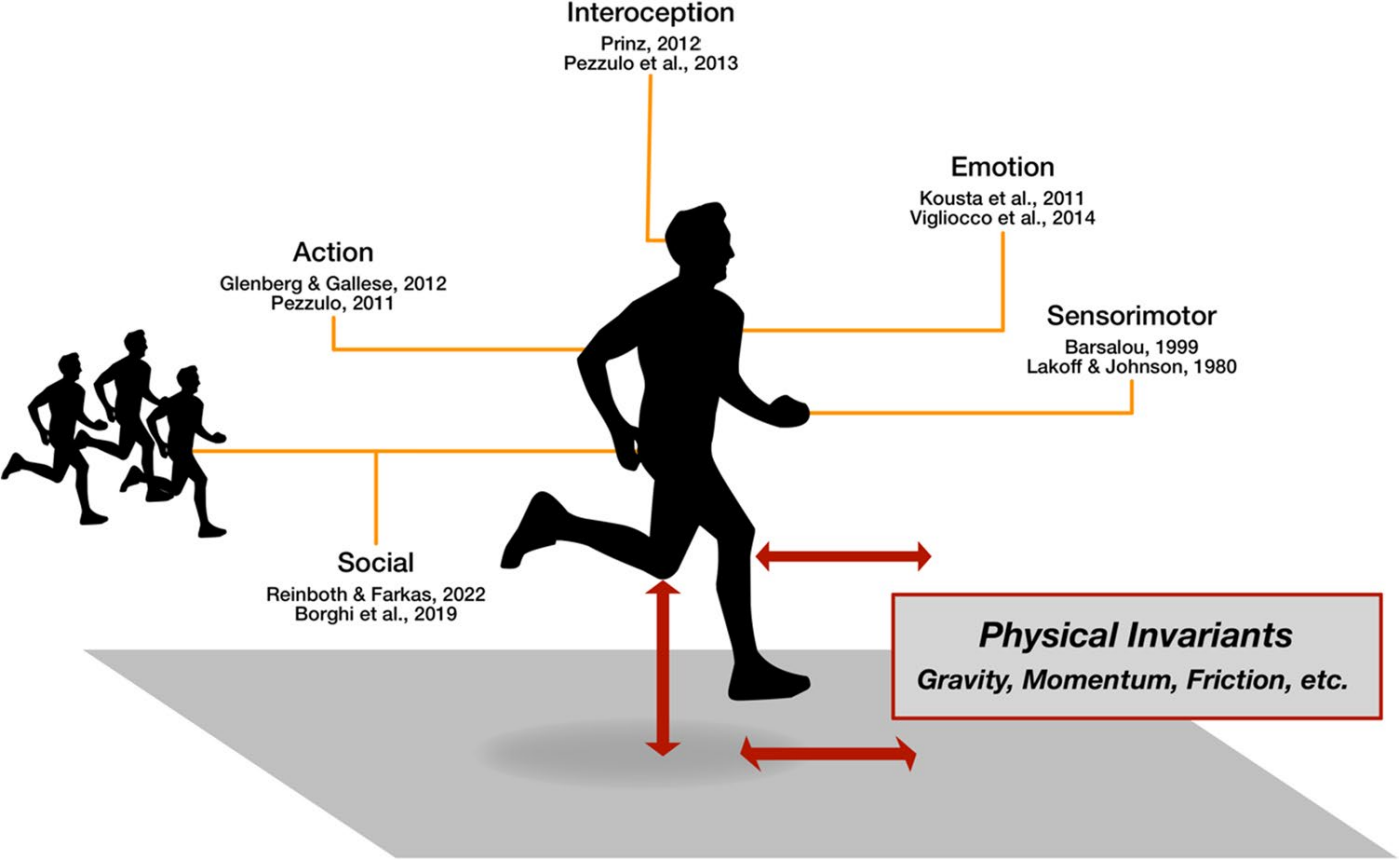
Modalities for grounding concepts. Our proposal (outlined in red) contributes a new source for grounding concepts: representations of physical invariants

We propose that the list of substrates in which to ground abstract concepts should be expanded to include invariant representations (see Fig. 1). In other words, we argue that kinetic force and kinematic direction are used to represent abstract concepts, and that the momentum of an object is similarly grounding this object’s mental representation as the warmth of its touch or its spatial location (e.g., up) in vision. The significance of this claim lies in its novelty and at the same time simplicity. The two central grounded cognition theories cannot accommodate it (although two others, conceptual spaces and predictive processing can; cf. below), despite the research summarized above clearly suggesting it. Physical invariants should long have been a grounding substrate as suggested by research on physical invariants and the two additional theories. Until now there is little empirical work explicitly stating this, and we are, to our knowledge, the first to propose this integration of invariant representations as a grounding substrate. Our argumentation therefore rests on synthesizing past research from diverse fields (see section *Previous and current research*), and arguing theoretically (section *Advantages of grounding in invariant representations*). While evidently extrapolating beyond past work, we are nonetheless confident that evidence is strong enough to make this bold claim.

To clarify, for the current (theoretical) discussion we refer to single modalities to preserve clarity. We also argue that *some* abstract concepts *can* be grounded, without meaning to say that all abstract concepts are grounded in concrete representations, or that they are necessarily all grounded in invariant representations. We solely argue that in the toolbox of representational substrates available to the conceptual system, there is a place for invariant representations.

## Theories of grounded cognition

There exists a wide range of theoretical positions describing the grounding or embodiment of knowledge. For current purposes we emphasize two main theoretical streams, simulation theory and conceptual metaphor theory, as these form the core of the grounded cognition discussion (as also done in, e.g., Lakens, 2014). Simulation is the most commonly cited mechanism in embodied cognition, and conceptual metaphor theory the most iconic and well-supported grounded cognition theory (Borghi et al., 2017; Gentsch et al., 2016). Furthermore, we look briefly at two further theories that are not as prolific in grounded cognition: Conceptual spaces and predictive processing. These theories are mentioned here because they are able to account for invariant representations in abstract concept grounding. For each of these four theories we discuss its core tenets and whether it acknowledges (or at least could account for) invariant representations as grounding substrate. The words as social tools approach (Borghi et al., 2019; Borghi & Cimatti, 2009) posits abstract concepts to be grounded in simulations and reflected in models of predictive processing. For this reason, we subsume it under our discussion of simulation theory and predictive processing. Another approach is the affective embodiment account, but this focuses much less on mechanisms and more on the importance of affect as grounding. We do not disagree with this proposal, but its discussion is not further pertinent to the topic of invariant representations.

### Simulation theory

Simulation approaches to cognition describe a perception- and action-based system underlying implicit memory, long-term memory, and conceptual knowledge (Barsalou, 2008, 2020). Its most elaborate version is *perceptual symbol systems*, which posits that, via selective attention, fragments of multimodal perceptions (called perceptual symbols) together form simulators, which in turn are the basis for cognition. On this view, “a concept is equivalent to a simulator” (Barsalou, 1999, p. 587). Concrete concepts consist largely of simulations of sensorimotor states, and abstract concepts largely of introspective or metacognitive representations, which include (partial) re-activation of mental states, cognitive operations, and emotional states (Barsalou, 2008). For example, to explain the concept of true, the experience of truth is simulated: a statement (“there is a dog on the table”) triggers a simulation (the cognitive state of seeing a dog on a table), which is then successfully compared with perception (looking at the table and seeing the dog on it; Barsalou & Wiemer-Hastings, 2005).

It is important to distinguish between two uses of the term “simulation”: Either as the name of a grounding mechanism (for current purposes: simulation mechanism) or as a name for Barsalou’s approach (simulation theory). The mechanism of simulation is usually used to describe any re-enactment of the states experienced during the perception of a concept (Körner et al., 2023), disconnected from the details of perceptual symbol systems theory. Perceptual symbol systems theory includes the mechanism of simulation, alongside details like simulators and convergence zones (as well as the mechanism of metaphor, most commonly associated with its rival conceptual metaphor theory; Barsalou, 1999, p. 600, see also Barsalou, 2020). Much work uses the term simulation, without explicating to what degree they adhere to mechanism or theory. This allows experimental work to avoid the theoretical baggage of perceptual symbol systems, but fails to explicate the degree of support for the theory.

Can invariant representations ground abstract concepts under simulation theory? Experimental work on one specific invariant, namely momentum (e.g., Freyd & Finke, 1984), is often mentioned in the context of simulation theory (e.g., Barsalou, 1999, 2009, Zwaan et al., 2004) although these mentions never argue that concepts are grounded in invariants. These references to simulations of momentum are merely used as evidence for a perception-based simulation system (as in the simulation theory from physical reasoning; Battaglia et al., 2013). In other words, although explicitly stating that simulation is the source of grounding, and using the simulation of invariants as evidence for this, the conclusion that it is possible to ground concepts in invariants has to our knowledge not previously been drawn.

Under simulation theory, abstract concepts are limited to being simulated in language, interoceptive and metacognitive states (Barsalou & Wiemer-Hastings, 2005; Pezzulo et al., 2013). Consequently, this theory would (implicitly) deny the possibility of grounding in invariant representations. Divorced from its theoretical baggage, the simulation mechanism may be able to ground concepts in invariant representations. The answer to this question would likely depend on whether kinetic force is internalized. If we assume that it is internalized, proponents of the simulation mechanism should acknowledge that it can be simulated and, therefore, qualify as a substrate for grounding.

Fischer and colleagues (Fischer, 2012; Fischer & Shaki, 2018; Myachykov et al., 2014; Pezzulo et al., 2013) have proposed a unifying taxonomy for the various mechanisms of grounding within the simulation framework, in which invariants are integrated into Barsalou’s (1999, 2008) work. They organized all influences that shape the grounding of concepts into three hierarchically related categories: situatedness, embodiedness, and groundedness. The hierarchical aspect of this theory comes from the notion that, in simulation, some sensory-motor aspects are more regularly present (e.g., the gravity vector) and others much more rarely (e.g., one specific orientation angle of your head). These sensory and motor constraints are considered to influence one another in the form of cascades (Pezzulo et al., 2013). At the most specific and influential level, *situatedness* describes the influence of current context. For example, turning your head to the left leads to better cognitive availability of smaller numbers in a random number generation task (Loetscher et al., 2008; Shaki & Fischer, 2014). The next level is *embodiedness*, referring to the role of the body and learned bodily constraints. For example, right-handers associate the right space with positive valence while left-handers associate the left space with positive valence as a result of previously experienced motor fluency (Casasanto, 2009). Lastly, *groundedness* (also called *tropism* in Myachykov et al., 2014), describes that, over millennia of evolution, the nervous system has become attuned to the environmental constraints over sensory and motor signals that shape our cognitive representations, as well as our bodily make-up.

Although applying physical invariants to grounded cognition, this hierarchical view does not explicitly suggest invariant representations as a grounding substrate. Therefore, it does not reflect the strong version of the present proposal in which invariants are a *direct* grounding substrate. Instead, physical invariants’ influences have more distal effects because they have shaped the biological system, and this in turn elicits cognitive biases through sensory and motor experiences. For example, evolution has elicited a hemispheric asymmetry associating lower numerosities with the left visual field and larger numerosities with the right visual field, thereby predisposing humans to adopt a population stereotype where small and large numbers become associated with left and right space, respectively (Felisatti et al., 2020; see also Vallortigara, 2018). Or consider the universal notion that “up is more,” which reflects the unidirectional influence of gravity, combined with the impermeability of objects, together resulting in the invariable experience that more objects pile up higher (M. H. Fischer, 2012; Fischer & Shaki, 2018; Lakoff & Johnson, 1980; Myachykov & Fischer, 2019). Therefore, this hierarchical framework does incorporate invariants, but merely as a basic constraint and not a grounding substrate.

### Conceptual metaphor theory

Conceptual metaphor theory is perhaps the most widely cited theory for grounding abstract concepts in concrete representations (Borghi et al., 2017; for a recent review, see Johnson, 2018; for a recent linguistics perspective, see Kövecses, 2020). It states that metaphors are not merely linguistic devices but expressions of the way the human conceptual system functions (Lakoff & Johnson, 1980). Abstract concepts are understood through the use of metaphors, which in turn are constructed of primary metaphors (primitive metaphors that underly many other, more complex mappings; Grady, 1997). These, in turn, are constructed of image schemas, structural patterns that are extracted from recurring perceptual patterns experienced through interaction with the environment (Johnson, 2018). In the language of conceptual metaphor theory, image schemas are the source domain (i.e., the concrete, grounding substrate) that is used to represent a target domain (i.e., the abstract concept). It is argued that “some (perhaps all) abstract reasoning is a metaphorical version of image-based reasoning” (Lakoff, 1990, p. 39). Despite conceptual metaphor theory’s strengths, especially regarding abstract concept grounding, there are also detractors that argue for significant issues in related empirical work (Gibbs, 2013; Pecher et al., 2011), and on theoretical grounds that the structural mapping would not suffice to represent concepts fully (Kövecses, 2008; Murphy, 1996; Pecher, 2018).

Unlike simulation theory, which grounds concepts in the simulation of the experience itself, conceptual metaphor theory says that concepts are grounded in image schemas (Gibbs, 2005, p. 90). For example, the image schema balance first originates from the proprioceptive experience of balancing the body, but is also later identified and applied when we have cold hands and warm them to restore a temperature balance, and then also experienced and deployed to represent balances of power or justice (example from Gibbs & Colston, 1995). Image schemas are manipulated to represent abstract concepts. For example, the image schema source-path-goal is used to structure the primary metaphor love-is-a-journey, from which we then derive conceptual metaphors like “we are at a cross-roads in our relationship” (Lakoff, 2012).

Does conceptual metaphor theory allow for grounding in invariant representations? This is difficult to answer, as concrete representations are not the grounding substrate under conceptual metaphor theory, but rather the already-abstracted image schemas (also see Gibbs, 2006). Nonetheless, two integrations of invariant representations and conceptual metaphor theory have been proposed. In the first proposal, invariants form image schemas (Gibbs, 2005, pp. 139–142). Therefore, invariant representations are themselves not the grounding substrate; instead, invariants (e.g., momentum) are so regular that they become image schemas themselves (e.g., momentum), or are an important factor in eliciting patterns that create other image schemas (e.g., SOURCE-PATH-GOAL; Gibbs, 2005; Gibbs et al., 2004; Gibbs & Colston, 1995). Johnson (2018, p. 628) explicitly states that gravity, via image schemas, is responsible for the verticality asymmetry expressed in “more is up” (cf. Fischer, 2012). However, this theoretical position denies the presence of kinetic content in the image schema, and therefore would not be reconcilable with a strong version of our proposal. This is not the case for the second integration.

The second integration, often clustered under conceptual metaphor theory, is Talmy’s (1988) *force dynamics*. The larger field of force dynamics is concerned with how forces (i.e., kinetic force) play a role in higher-level cognitive domains, such as perception of social dynamics (Wolff, 2017). The proposal by Talmy (1988) argues that image schemas consist also of kinetic force, and are not reducible to just structural or image-schematic relations. It further emphasizes that concepts can gain meaning by their interrelations, and frames these in terms of agonists and antagonists (Talmy, 2000). The theory contributes to understanding language and language structure, but does not intend to explain mental representations of concepts. Talmy’s view is, to date, the theory closest to our proposal of invariant representations, but it has not received much acclaim beyond being cited as a historical piece. Despite this, it is mentioned in many texts central to grounded cognition like Barsalou (1999), Gallese and Lakoff (2005), and Gibbs (2005), which is indicative of its significance. Yet these texts do not build on Talmy’s work beyond its reference to image schemas (continuing to avoid kinetic force), in part because the work is focused more on language and does little to illuminate the nature of mental representations. Furthermore, relevant for the current proposal is that Talmy’s force dynamics proposes that representations of kinetic force are included in image schemas. As image schemas are already abstracted from perception, force dynamics is not in line with the strong version of our proposal. Therefore, for current purposes, Talmy’s work is symbolically important because it was the first (and to our knowledge only) impactful work in grounded cognition to explicitly emphasize kinetic force, but beyond this, it does little to contribute to explaining concept grounding.

As with the simulation theory and the simulation mechanism, conceptual metaphor theory is often implicitly equated with the mechanism of metaphor. Conceptual metaphor theory uses the mechanism of metaphor, but attaches various constructs like image schemas and primary metaphors. The metaphor *mechanism* is just the representation of an abstract domain by reusing a different, more concrete representation (Casasanto & Gijssels, 2015; Jamrozik et al., 2016). The mechanism, despite being frequently cited as a critical component of grounded cognition, is vastly underspecified (Desai, 2022).

### Further perspectives

Although simulation and conceptual metaphor theory are the primary theories of grounded cognition, we present two further approaches that we believe have significant merit in the current discussion. *Conceptual spaces* and *predictive processing* are both able to explain the function of invariant representations in grounding abstract concepts. Viewing grounded cognition through their perspective gives novel impulses for progressing grounded cognition. Both are uniquely positioned to account for invariant representations in cognition, which makes their proposals interesting to grounded cognition research.

### Conceptual spaces

Conceptual spaces theory says that concepts are represented in mental spaces (for an introduction, see ch.1 of Zenker & Gärdenfors, 2015). Although arguably not an embodied theory, because it does not emphasize the body in representing concepts, it says that concepts are represented by being associated in a geometrical, multi-dimensional space. The dimensions that make up this geometrical space are called “quality dimensions” (i.e., attributes on which concepts differ, e.g., for cars their speed, size, or redness) and are often derived from sensory experience, but not necessarily so (Gärdenfors, 2014). A single concept is a region within this space, with more similar concepts being closer than dissimilar concepts (Zenker & Gärdenfors, 2015). The representation of, for example, flavor, exists on the different quality dimensions sweetness, bitterness, sourness, etc. and any taste is represented by being positioned in this region (Gärdenfors, 2000). Conceptual spaces states that abstract concepts gain meaning through metonymy (refocusing attention, irrelevant for current purposes) and through the metaphor mechanism (Gärdenfors, 1996). The latter in this case involves re-applying the geometrical (conceptual) structure from source domains to represent the structure of abstract domains (Gärdenfors, 2014). Abstract concepts are therefore represented using the same spatial representations as concrete concepts. Furthermore, abstract concepts are also represented in such spaces, but then typically on quality dimensions that are not sensory in nature (Gärdenfors, 2000, 2014).

Do invariant representations play a role in conceptual spaces theory? The conceptual space and its qualities are shaped by environmental constraints, including physical invariants (Gärdenfors, 2014, p. 22). Gärdenfors emphasizes that invariants are internalized (Gärdenfors, 2020, 2021), and thus present in the conceptual spaces. Physical invariants play a role in conceptual spaces by similarly shaping movement; just like movement in the environment involves momentum, there also exists momentum in conceptual space. Consequently, they play a constitutive role for concept representation (Gärdenfors, 2007, 2014, p. 33), and this view has even been reconciled with Talmy’s (1988) force dynamics (Gärdenfors, 2007). Further, kinetic force is not only deployed in representing concrete concepts, but also in abstract concepts (Gärdenfors, 2007, 2014, p. 91; see also Gärdenfors, 2021). Therefore, kinetic force plays a role in the representation of abstract concepts in two ways: Firstly, because of its presence in the conceptual space generally. Abstract concepts that are represented on non-sensory quality dimensions would nonetheless be affected by invariants because kinetic force is generally present in the conceptual space. And secondly, through metaphor, which would reuse the representation of kinetic force. In this sense, conceptual spaces provides a unique incorporation of physical invariants, allowing for a strong version of our proposal by providing two mechanisms for grounding in invariant representations, and by embedding this proposal into a larger theory of concept grounding. Therefore, future research assessing physical invariants would be well helped by referring to past work on conceptual spaces.

### Predictive processing

Predictive processing is a more general and unified account of perception, cognition, and action, based on the principle of predictive coding (Clark, 2013; Hohwy, 2020). Predictive processing states that forward models filter incoming sensory signals. In a hierarchical structure, with the highest level being the most abstract and general, and the lowest the most specific, many levels each consist of models that predict the level below. For example, a high-level model consisting of a friend calling me on my cell phone provides predictions for the level below, that there is a familiar melody ringing from my phone; this elicits even more specific predictions in the lower levels, such that I hear noise and that the rectangular spot in my right visual field is lighting up. If each model is correct, there is no discrepancy between the models’ predictions and the incoming sensory signal. It may be incorrect though, and the noise I am hearing is my radio alarm, in which case the prediction of a rectangle in my visual field lighting up is violated and an error signal travels up the hierarchy. A new model is created, whose predictions cascade down the hierarchy. This prediction system is constantly being updated and is always aiming to minimize prediction errors (Friston, 2010).

Under this view, the forward models (except for the first) can be viewed as representations (Clark, 2015), with increasing levels corresponding to increasing levels of abstraction (Gilead et al., 2020; Tenenbaum et al., 2011). Importantly, models can be detached, i.e., allow for covert simulation, allowing representation of objects in their absence via internal loops (Grush, 2004; Pezzulo, 2011; Wolpert et al., 2003). What is typically called a “concept” in other cognition literature, would here be the equivalent of a high-level model (Van Elk & Bekkering, 2018). The general framework under which to assess representation under predictive processing is often characterized under a theoretical account called structuralism^5^ (Gładziejewski, 2016; Hohwy, 2013). This account emphasizes a resemblance between the external and the mental world (O’Brien & Opie, 2004, 2015). This resemblance is not a 1-to-1 mapping; instead, the relations between objects in the external world correspond to the relations of the internal world, as a map reflects the structure of a territory (Gładziejewski, 2016). This structurally isomorphic model is also called a “world model,” and is used to perceive, act, and represent concepts (Friston et al., 2021; Pezzulo et al., 2023).

Regarding abstract concepts and what substrate they are represented in, predictive processing accounts of concepts emphasize the motor system (Gallese & Metzinger, 2003; cf. action oriented representations; Pezzulo, 2017), and metaphor. Covert action-models can model abstract relations: grasping a cup relies on the same models as grasping an idea (Pezzulo, 2011, p. 102). Language generally provides models with which to represent concepts (Lupyan & Clark, 2015). Abstract concepts often do not have single concrete referents, and language, by its arbitrary nature, provides a single label that “carves joints into nature” (Lupyan, 2012; Lupyan & Winter, 2018). The “words as social tools,” based on a simulation and predictive processing account, views words as tools that change the physical and social state of the world (cf. Clark, 1998b). Under this view, concepts are grounded in predictions of such changes (Borghi et al., 2013, 2019).

Importantly, the existence of invariant representations is assumed under predictive processing (Friston, 2008). Indeed, it is well acknowledged that we can generate internal models of experiences that we have never produced ourselves, like the roll of a wave (Schubotz, 2007; Wolfensteller et al., 2007), or as we argue, momentum and gravity. Within predictive processing research, it is not a point of contention that “gravity is encoded in a robust forward model” (Torricelli et al., 2022, p. 33). Much work has described a robust model for gravity (Zago et al., 2008; Zago & Lacquaniti, 2005), especially from the field of physical reasoning as described above (Battaglia et al., 2013; Pramod et al., 2022). Torricelli et al. (2022) go even further, stating if gravity is encoded as a forward model, “motor invariants” (regularities in *motor behavior*) should also be used in perception and cognition. It is also argued that the models are not mere reflections of kinematics, but rather kinetic force as well (Lacquaniti & Zago, 2001), motivating a strong version of our proposal. In short, abstract concepts are represented via metaphor (using models originally developed for action to represent abstract concepts), and predictive processing emphasizes the role of physical invariants. Consequently, therefore, abstract concepts could be grounded in physical invariants in predictive processing via metaphor, although application of these invariants to abstract concept representation has not been elaborately developed. With work on predictive processing clearly evidencing that even abstract cognition consists of models from experience with the world, and models of gravity being explicitly proposed, it is overdue to acknowledge the role of grounding in invariant representations under predictive processing.

## Invariant representations in concept grounding

Until here, we provided an overview of invariant representations and of grounded cognition, followed by an introduction to various grounded cognition theories and their (explicit or implicit) commitments to grounding in invariant representations. Next, we compile this work, beginning with a summary of the current status of invariant representations in grounded cognition, followed by a look at the scarce empirical work on grounding in invariant representations. After this summary, we look ahead to the consequences of invariant representations for the human conceptual system and for the grounded cognition research program.

### The current status of invariant representations in grounded cognition

There is considerable evidence for the mental representation of physical invariants and its component kinetic force, as described in the work on causal perception and physical reasoning above. We argue that this should make them a candidate grounding substrate because the core tenet of grounded cognition is that the brain uses concrete (i.e., experiential) representations to represent even abstract concepts. With diverse grounded cognition theories proposing different mechanisms, we next assess whether and how the theories described above acknowledge grounding in invariant representations.

A wide variety of modalities have been posited for the grounding of cognition, but kinetic force has been excluded until now. The list of modalities comprises sensorimotor states (Barsalou, 1999; Lakoff & Johnson, 1980; Speed & Majid, 2019), action-based states (Glenberg & Gallese, 2012; Pezzulo, 2011), proprio- and interoception, metacognitive states (Barsalou & Wiemer-Hastings, 2005; Pezzulo et al., 2013; Prinz, 2012), social interactions (Borghi et al., 2019; Reinboth & Farkaš, 2022), and emotions (Kousta et al., 2011; Vigliocco et al., 2014). As none of the grounded cognition theories reviewed above includes the simulation of kinetic force, we conclude that no current formulation of a grounded cognition theory includes kinetic force or invariant representations. The closest is the theory of conceptual spaces, which is rarely mentioned in the grounded cognition discourse, but which makes explicit that invariant representations (including their kinetic force) play a constitutional role in concrete as well as abstract concept grounding.

A few passing mentions of grounding in a “force modality” have been made in past literature. It is absent from most reviews of the embodiment literature, and when it is mentioned it is as an aside or a historical artifact (e.g., Borghi et al., 2017; Pecher et al., 2011), although this absence has been mentioned (Gärdenfors, 2014). The only theory connecting grounded cognition and kinetic force is force dynamics (Talmy, 1988), which has so far not been included in recent work on grounded cognition. A short mention of grounding in invariant representations can be found in Johnson (1987). Furthermore, Lakoff and Johnson (Lakoff, 2012; Lakoff & Johnson, 1999) also mention “force type cogs” (i.e., image schemas based on forces such as pushing force) based on Talmy’s work, but these image schemas only consist of spatial relations and do not contain kinetic content.

Our review of four different theories above draws a picture in which the two classic theories of grounded cognition pale in comparison to both conceptual spaces and predictive processing in their capacity to integrate invariant representations, and this state of theorizing motivated our present proposal. Neither simulation nor conceptual metaphor theory acknowledge invariant representations or grounding in kinetic force. Talmy’s force dynamics does, but its focus is more on force schemas, which are already abstracted representations. Conceptual spaces theory, on the other hand, is a theory that at its core integrates kinetic force and physical invariants into the conceptual system (Gärdenfors, 2014). Conceptual spaces theory has been reconciled with conceptual metaphor theory and force dynamics (Gärdenfors, 2007, 2014, p. 72), and is readily integrated into other domains of cognitive science, such as computational approaches in neuroscience (Kriegeskorte & Kievit, 2013), forward modeling approaches similar to predictive processing (Gärdenfors, 2004; Michel, 2020), and spatial cognition (Bellmund et al., 2018). Predictive processing has also taken physical invariants into account, going even further by also positing motor invariants (Torricelli et al., 2022). Internal models of gravity are well evidenced (Battaglia et al., 2013; Zago & Lacquaniti, 2005), and predictive processing’s representational account of structuralism posits a “world model” that is used to simulate concepts (Williams, 2018). The tenability of grounding under these theories is further bolstered by the “words as social tools” approach, which joins predictive processing and similarly extends the sphere of “grounding” beyond the body (Borghi et al., 2019). Therefore, both conceptual spaces and predictive processing views can accommodate invariant representations in the representation of concepts, with the former already demonstrating success in this endeavor.

The elaborate theories of simulation and conceptual metaphor theory have not mentioned and do not allow for invariant representations. However, when stripped down to their mechanisms, they could accommodate invariant representations well, as the simulation mechanism would treat kinetic force as “just another modality” that grounds concepts by simulation. This same argument holds for conceptual metaphor theory: Under its elaborate theory version, invariant representations can shape image schemas, but kinetic force is excluded because image schemas only consist of spatial relations. Yet, again, when stripping the theory of its theoretical baggage, and looking only at the mechanism of metaphor, invariant representations would be a powerful addition to concept grounding approaches.

### Previous and current research

To assess the tenability of our proposal, we now look at past empirical research. Perhaps the closest theory to ours is the *environmental invariants hypothesis* (Hubbard, 1999), which we briefly introduce next. Although this theory does not explicitly address grounded cognition, it similarly proposes that representations of invariants are redeployed for abstract domains. Following this, we examine past research through the lens of invariant representations. Past research on kinematics may have been inadvertently assessing invariant representations, and we consider whether invariants may have been responsible for patterns of experimental results.

### An existing account – the environmental invariants hypothesis

The aptly named *environmental invariants hypothesis* states that physical invariants are not only deeply engrained as building blocks of perception, but that they are reused for a variety of concrete and abstract domains (Hubbard, 1999). The hypothesis originates from work on the *representational momentum effect*. Here, moving objects that are suddenly occluded are perceived as further along in their direction of movement than their actual location at occlusion (Freyd & Finke, 1984; Hubbard, 2005; Merz, 2022). This bias has been argued to be caused by an internal simulation of the object’s movement, which includes the kinetic force of momentum (the internal version of momentum being called *representational momentum*) acting upon it (Hubbard, 2010). Similar findings were later reported for other invariants: *representational gravity* (objects are perceived as lower than their actual location), *representational friction* (such effects are reduced when objects move on a surface), and *representational centripetal force* (objects are perceived as moving away from the center of a circular movement; see Hubbard, 1995, 2020). Importantly, these effects go beyond artefacts of visual perception: They hold also for auditory stimuli, where an overshoot obtains after increase in pitch, resulting from perceiving “momentum” (Freyd et al., 1990; Johnston & Jones, 2006; for a review of physical motion in music, Hubbard, 2017). Similarly, this overshoot also exists for increasing luminance (Brehaut & Tipper, 1996), changes in state such as melting ice (Hafri et al., 2022), and in tactile movement (Merz et al., 2019, 2021). These latter observations strongly suggest that invariant representations go beyond superficial extrapolations and consist of complex multi-modal representations including kinetic force. The invariant representations are not just applied throughout vision but are also used to perceive, understand, and structure other modalities.

The viability of our argument is further strengthened by the abundant presence of invariants in other, even more abstract, domains of perception and cognition. For example, Markman and Guenther (2007) found evidence for psychological momentum (improved performance after a sequence of successful actions) being based on the same mechanisms as representational momentum. They found that mass, a factor that influences both physical and representational momentum, exhibited a similar role in modulating the effect of psychological momentum when predicting the behavior of a fictitious person. Similarly, attentional momentum, i.e., an overshoot when moving attention from one display location to another, is also suggested to be a part of the class of representational momentum phenomena (Spalek & Hammad, 2004). Lastly, operational momentum, overestimation during addition and underestimation during subtraction tasks, is often suggested to be the result of overshoot along the “mental number line” (Knops et al., 2009; McCrink et al., 2007; see also Shaki et al., 2018; Mioni et al., 2021). Perhaps even stronger support for concept grounding in the component of kinetic force especially, is the recent observation of corresponding changes in grip force by participants thinking of higher numbers (more force) or lower numbers (less force; Miklashevsky et al., 2021, 2022). Further evidencing the role of invariants specifically, the perception of gravity impacts random number generation such that a supine body position leads to generating smaller numbers than upright (M. Gallagher et al., 2019). Overall, the environmental invariants hypothesis has shown considerable empirical evidence for a multi-modal representation of invariants, including kinetic force, which is recruited in service of abstract cognitive abilities (for a more holistic and atheoretical account integrating physical invariants and higher-level cognition, see Kent, 2024).

### Past research through the lens of invariant representations

The proposal of concept grounding through kinetic force has, to our knowledge, only been directly tested once in an unpublished study. We describe it here to illustrate possible testing in future work. Madden and Pecher (2007; reported in Pecher et al., 2011) found support for force schemas in grounded cognition using an interference paradigm. They asked participants to judge the sensibility of sentences that included kinetic force, in either concrete settings (e.g., The bulldozer pushed the pile of dirt across the lot) or abstract settings (e.g., Her friends persuaded the girl to come to the party). Prior to making these judgments, participants saw an animation that presented two shapes interacting according to a certain force schema (e.g., a circle causing a rectangle to topple). When the animation activated the same force schema as the subsequent sentence, the sensibility ratings were quicker and more accurate. Importantly, in support of the strong version of our proposal, this pattern held for concrete as well as abstract sentences (Pecher et al., 2011), suggesting that kinetic force is used not only to ground concepts, but also to ground abstract concepts. It should be mentioned that this research has not been peer reviewed, its results were somewhat inconsistent, and these could also be explained by alternative mechanisms like visual priming.^6^

Apart from this study, one can also re-examine other “embodied effects” in the current literature. We aim to show that a number of results could be parsimoniously explained by invariant representation. Nonetheless, these are post hoc interpretations on published data, and therefore should be viewed with the necessary caution. Generally, a large number of “embodied effects” is concerned with an asymmetry in cognition based around the vertical axis: up is good, more, powerful, moral, etc. and vice versa (Casasanto & Bottini, 2014; Lynott & Coventry, 2014; Meier & Robinson, 2004). The same asymmetry holds along the horizontal axis: right is more, good, forward, future, or progress, and vice versa (Fischer & Shaki, 2018; Malyshevskaya et al., 2023; Weger & Pratt, 2008). These kinematic direction biases are often described as the result of cognitive (as opposed to environmental) universals, such as a tendency to express positive emotions with upward bodily movement (raising your hands in success, or sitting upright in pride), of making progress by moving forward (Lakoff & Johnson, 1999), or of a generalized tendency to identify and ascribe polarity orientations of + and – to objects, directions, regions, etc. (Lakens, 2012; Proctor & Cho, 2006). We argue that both vertical and horizontal axes could be viewed through the lenses of gravity and momentum, respectively, where these embodied associations do not stem from the motor system, but rather from invariant representations’ associated kinematic directions.

In support of this view, we present here two studies that may have inadvertently assessed invariant representations. In the first study, participants gave higher judgments of importance when they held a heavier compared to a lighter clipboard (Jostmann et al., 2009). This result replicated when the heavy item was an unrelated shopping bag (Zhang & Li, 2012) as well as when the judgment was of one’s own memory (Alban & Kelley, 2013). While a classic embodied account would argue that the experience of weight is associated with importance, we argue that heaviness also primes the concept of gravity and thus an invariant representation. Indeed, Jostmann et al. (2009) even argued that this effect might have its origin in the invariant nature of gravity.

The second example comes from Richardson et al. (2001), who assessed how verbally described interactions between two agents are visually depicted through image schematic relations. The described interactions were either concrete (e.g., A pushes B) or abstract (e.g., A respects B) relations between the two agents. Participants related two shapes (A and B) by an arrow horizontally left to right, horizontally right to left, vertically up, or vertically down (study 1) or placed shapes and arrows freely in a visual editor (study 2). The authors found consistent choices among participants not only for concrete, but also for abstract relational concepts. For example, the description “A wanted B” was chosen by 61.1% of participants as a horizontal arrow going from left to right (i.e., A to B). While the authors conclude that this result reflects a universal image schema, they cannot preclude the alternative explanation that the underlying representational substrate is kinetic force. A participant’s choice in drawing an arrow from A to B may have reflected a kinetic force drawing A towards B. None of the studies mentioned in this section relied on invariant representations in their theoretical reasoning, but we argue that this would be a fitting explanation. Nonetheless, this is post hoc theorizing, and therefore we cannot make strong claims as to whether these explain data better than the original hypotheses.

### Advantages of grounding in invariant representations

What does a conceptual system have to gain by including invariant representations in its toolbox? It is critical for a grounding substrate to be flexible (e.g., language is an abstract concept grounding substrate for this reason), and the same can be said of invariant representations. Invariant representations are experienced in a vast number of different forms (e.g., momentum can go in any direction, and gravity interacts with momentum to elicit infinite new paths that objects can move along; friction brings a cube to stop and a ball to roll), and their experiences could be simulated flexibly. Despite this, and unlike language, such simulations of the real world maintain a wealth of semantic information (O’Brien & Opie, 2015). Not to mention that kinetic force arguably adds another modality, augmenting the grounded conceptual system. Much like the richness that interoceptive representations contribute as a grounding source (Barsalou & Wiemer-Hastings, 2005), invariant representations and kinetic force may do so as well.

More generally, what does the grounded cognition research program have to gain by including invariant representations in its theories? Firstly, because invariants are universal, they do not depend on situational factors and may therefore explain cross-cultural regularities. With culture and different languages being increasingly in focus in grounded cognition (e.g., Barsalou, 2023; Kemmerer, 2022; Majid, 2023; Thompson et al., 2020), the notion of a universal, which invariant representations would present, could form an important complement to culture-, location-, or person-specific principles. Second, our proposal could compensate for past weaknesses in strongly embodied grounded cognition accounts. For example, Muraki et al. (2023) recently assessed to what degree concepts are simulated by studying persons who cannot engage in mental imagery (so-called aphantasiacs). Their inability to perform mental imagery should correspond to an inability to represent some concepts. Our proposal argues that the ability to ground concepts in kinetic force would compensate for difficulties originating from aphantasia. When grounding concepts is not limited to the motor or sensory system, results such as maintained object simulation by aphantasiacs could be parsimoniously explained.

What does cognitive science generally gain by including invariant representations? The novelty of implications emerging from invariant representations should not be understated. Since the grounding problem in standard symbol manipulation approaches to cognitive science was forcefully identified (Harnad, 1990; Searle, 1980), conceptual grounding is typically assumed to be an *embodied* process, so that any modality in which concepts are grounded is a perception of the *body* (touch, action, interoception, emotion, etc.). Invariant representations are representations of features outside the body, and therefore beyond the implicitly assumed outer limit of grounding. This constitutes a drastic expansion, and its implications can be demonstrated by looking at prior terminology in grounded cognition work. Some discussions of embodied and grounded cognition use the distinction of amodal versus bodily formats (e.g., Cuccio, 2014; Gallese, 2017; Hauke et al., 2016; cf. Goldman & de Vignemont, 2009). This terminology demonstrates how strongly grounded cognition approaches implicitly assume that concepts can only be grounded in representations within the body (or of the body’s interaction with others; Borghi et al., 2019). A non-bodily, but still experiential, format of physical invariants that nonetheless grounds the conceptual system would overturn this assumption and push grounded cognition to a more extended conception of the mind (for a similar idea see, Borghi et al., 2013, 2023).

### Invariant representations – an outlook

Having discussed the status of invariant representations in grounded cognition, we see four questions that future research on invariant representations should address:

First, how could invariant representations ground concrete concepts? Theories of grounded cognition often state that based on their attributes, concrete and abstract concepts require different grounding substrates (Lynott et al., 2020). Invariant representations could well ground concrete concepts via simulation, alongside sensorimotor representations. As explained above, for the example cup this may be the sensory experience of holding a smooth or warm cup. In the case of physical invariants grounding concrete concepts, we argue that concepts are grounded by simulating the kinematic and kinetic components of the represented concept. For example, the kinetic force and kinematic direction of gravity acting on a cup, pulling it downwards.

Second, how could invariant representations ground abstract concepts? Abstract concepts are more complex, emotional, or variable when compared to concrete concepts (Desai et al., 2018; Villani et al., 2019), thus requiring more of the rich visceral information afforded by, for example, interoception or emotion (Barsalou & Wiemer-Hastings, 2005; Connell et al., 2018; Vigliocco et al., 2014), or by the flexibility of language (Andrews et al., 2009; Dove, 2011; Lupyan, 2012). Therefore, invariant representations, by their nature of being physical and tangible, are likely suited most to grounding abstract concepts only via the mechanism of metaphor. Similarly, it has been argued that a series of successes when one finishes multiple tasks in sequence, evokes the experience of the physical invariant momentum (Markman & Guenther, 2007). These examples involved the mechanism of metaphor (without the theoretical elaborations of conceptual metaphor theory). Yet, it is perhaps premature to limit invariant representations’ grounding potential to the metaphor mechanism. As explained above (section *Advantages of grounding in invariant representations*), invariant representations are at the same time more flexible, not being bound to the body, and more experientially rich, as afforded by abstract concepts. The many important ways in which invariants differ from bodily sensations could therefore make invariant representations a powerful grounding mechanism for abstract concepts especially. This is the way in which conceptual spaces argue for grounding in invariants (although we, again, do not need to limit ourselves to this interpretation), where physical invariants such as momentum act in the conceptual space of abstract quality dimensions (Gärdenfors, 2007, 2014). An example of this could be found in music, where the perception of musical motion and forces draws on the same representational substrate as the perception of kinetic force itself (Hubbard, 2017; Larson, 2012).

Third, what past effects in grounded cognition research were caused by the kinematic component of invariant representations? Many findings in support of classical sensorimotor grounding could be explained just as well, or better, with invariant representations (see section *Past research through the lens of invariant representations*). Furthermore, non-replication of some of these studies could be explained by the mechanism being kinetic force, which was then perhaps modified in the replication attempt. This would be the case if an original study conflated kinematic direction and kinetic force while the replication only manipulated kinematic direction. One example where a replication failure likely changed the kinetic component of responses from the original method is Morey et al. (2022). They re-ran the foundational study of Glenberg and Kaschak (2002) that discovered the action sentence compatibility effect, which is key evidence for the simulation of sentence meaning. However, while the original study required directional movements with consistent mappings in every trial, the replication used a go/no-go task where participants decided whether to move on every trial. Moreover, movement mappings changed randomly across trials. Both deviations from the original method increased response uncertainty, which likely led to reduced response forces (Balota & Abrams, 1995). This could explain the failure to replicate the original finding (cf. Körner et al., 2023).

Fourth, and avoided until now, is the question about the origin of invariant representations. These representations are likely shaped by the dedicated features of the vestibular system. The utricle and saccule contain fine hairs that respond to even very small kinetic forces acting on the body (Berthoz, 1996; Khan & Chang, 2013). Embodied cognition approaches would state that we learn about invariants via interaction with objects. Having picked up many things using our arms, we learn about gravity, a representation that is then later redeployed when viewing objects moving (Johnson, 2018; cf. Kirchhoff & Kiverstein, 2019, p. 94ff). Another position could be that the body is not necessary and that purely visual information suffices to acquire the concept of gravity. Having often enough seen unsupported objects fall, and supported objects to not fall, gravity is at first inferred and then later represented directly (similar to “core knowledge” approaches; Mandler, 1992; Spelke & Kinzler, 2007). Thirdly, it is also possible that kinetic force is first experienced visually, and later represented through the body. There has been work demonstrating that viewing inanimate movements, like a rolling wave, also elicits activation of the motor system (Schubotz, 2007; Wolfensteller et al., 2007), and that sensorimotor representations are recruited even when learning concepts solely via language (Günther et al., 2020). Lastly, most plausible is a combination of both positions: that both visual and motor competencies develop, supporting and informing each other, producing a multi-modal representation of kinetic force. On a logical note, that the brain would infer kinetic force should also not be a controversial position: The brain infers space, distance, and shapes from the pattern of light which arrive at the retina, and it is not controversial to argue that these are ‘perceived’ (Gärdenfors, 2004). Based on this literature, we feel comfortable in assuming that the pre-requisite for invariant representations to qualify as a ‘grounding substrate’ is met. 

What we have formulated here is the strongest possible claim, namely that *all components* of invariant representations can be *direct* grounding substrate for *abstract* concepts. We invite future work to falsify this claim in its first, second, and third parts (see italics). Firstly, the claim can be modified in that that concepts can only be grounded in the kinematic component of invariants. The contribution would still hold that invariants are a grounding substrate. A second way to modify this claim is that invariants are not necessarily the direct substrate in which concepts are grounded, but are distal mechanisms which constrain and cause grounding effects. This would align our proposal with perspectives presented by Fischer (2012; cf. also Felisatti et al., 2020), Gibbs (2005, p. 141), and Johnson (2018), although the latter two do not place importance on physical invariants. Third, one can also modify the claim so that it grounds only concrete concepts. Even when assuming that invariants are a grounding substrate because they are simulated as well as other modalities, they may still be limited to concrete concepts, as has been purported for sensorimotor representations (Barsalou & Wiemer-Hastings, 2005; Borghi et al., 2019; Kousta et al., 2011). Lastly, in addition to these three possibilities, it is also possible to modify the claim by arguing that invariant representations are already “abstracted” perceptions (cf. Merfeld et al., 1999), yet can still contribute to grounding concepts (as in dynamic force image schemas). All these weaker formulations of our proposal would still be novel and interesting contributions to the ongoing grounded cognition work by explaining the origin of grounded effects, or by giving a framework in which to embed other findings. We invite falsificationists to whittle away the untenable parts of our strong version.

## Concluding remarks

We have outlined a proposal that invariant representations can be a direct grounding substrate for abstract concepts. We have also reviewed the grounded cognition literature, finding that the central theories (simulation and conceptual metaphor theory) are unable to account for physical invariants in their elaborate versions. Meanwhile two theories less commonly cited in grounded cognition literature, conceptual spaces and predictive processing, are well able to account for all of our strong proposal (conceptual spaces) or very large parts (predictive processing). We conclude that invariant representations deserve to be considered as a grounding substrate. Finally, we have argued that it would be beneficial for grounded cognition literature to differentiate simulation and metaphor as theories or as mechanisms (i.e., without their corresponding theoretical additions). In this reduced mechanism-version of the theories, physical invariants could be valuably integrated into current grounded cognition literature. This account begets an assessment of invariant representations and is well positioned to be integrated into current discussions of cognitive science and beyond (such as artificial intelligence).

## Data Availability

There is no data available because it is a theoretical review. We did not perform any analyses and used no data.

## Footnotes

1 We use the term physical invariants (1) to align, and enable integration, with the term motor invariants (from Torricelli et al., 2022) and (2) because the alternative term, environmental invariants (used by, e.g., Hubbard, 1999) is too broad, being a term for all unchanging features of the environment. This goes beyond the physical, for example, that time only progresses forward, and is therefore not the focus of the current paper.

2 Past literature in cognitive science has often used the term “force” (e.g., White, 2012) or a variant of dynamic (e.g., “dynamic” in Cohen, 2006; “internalized dynamics” in Bertamini, 2002; or “force dynamics” in Talmy, 1988) to describe this component. Yet, because “force” is non-specific and “dynamic” is used to describe an evolution over time, especially in cognitive science where the term is connected to dynamical systems theory (e.g., Kelso, 1995; Port & Van Gelder, 1995), we prefer the term “kinetic,” which is unlikely to be conflated with another, is sourced directly from classical mechanics, and has a counterpart “kinematic.”

3 Not to be confused with Barsalou’s (1999, 2008) simulation theory below. We imply that this is not a mere coincidence and these theories reflect the same mechanism, but the authors named here may not.

4 For a classic review on grounded cognition, see Barsalou (2008); for a more recent review, see Muraki et al. (2023); focused on abstract concepts, see Borghi et al. (2017) or Pecher et al. (2011); focused on neuroscience, see Meteyard et al., (2012) or Bechtold et al. (2023); for shortcomings, see Pecher (2018; Pecher & Zeelenberg, 2018); and for criticisms of the field, see Goldinger et al. (2016) or Mahon and Caramazza (2008).

5 The representational commitments of predictive processing have been fervently debated. We align with Gładziejewski (2016; Gładziejewski & Miłkowski, 2017). See Michel (2020, 2022) for an alternative view, and Downey (2018) or Facchin (2021) for counterpoints.

6 We thank Diane Pecher for pointing this out during the review process.
